# Modeling patients’ time, travel, and monitoring costs in anticoagulation management: societal savings achievable with the shift from warfarin to direct oral anticoagulants

**DOI:** 10.1186/s12913-019-4711-z

**Published:** 2019-11-27

**Authors:** Aapeli Leminen, Mikko Pyykönen, Juho Tynkkynen, Markku Tykkyläinen, Tiina Laatikainen

**Affiliations:** 1 Department of Geographical and Historical Studies, University of Eastern Finland, P.O. Box 111, 80101 Joensuu, Finland; 2 Faculty of Medicine and Health Technology, Tampere University, Kalevantie 4, 33100 Tampere, Finland; 3 Department of Radiology, Kanta-Häme Central Hospital, Ahvenistontie 20, 13530 Hämeenlinna, Finland; 4 Institute of Public Health and Clinical Nutrition, University of Eastern Finland, P.O. Box 1627, 70211 Kuopio, Finland; 5 Joint municipal authority for North Karelia social and health services, Tikkamäentie 16, 80210 Joensuu, Finland; 6 Department of Public Health Solutions, National Institute for Health and Welfare (THL), P.O. Box 30, 00271 Helsinki, Finland

**Keywords:** Atrial fibrillation, INR monitoring, DOAC, Real-world data, Network analysis, Accessibility, GIS, Cost model

## Abstract

**Background:**

Anticoagulation therapy is used for atrial fibrillation (AF) patients for reducing the risk of cardioembolic complications such as stroke. The previously recommended anticoagulant, warfarin, has a narrow therapeutic window, and it requires regular laboratory monitoring, unlike direct oral anticoagulants (DOAC). From a societal perspective, it is important to measure time and travel costs associated with warfarin monitoring to better compare the total therapy costs of these two alternative forms of anticoagulation management. In this study we design a georeferenced cost model to investigate societal savings achievable with the shift from warfarin to DOACs in the study region of North Karelia in Eastern Finland.

**Methods:**

Individual-level patient data of 6519 AF patients was obtained from the regional patient database. Patients’ geocoded home addresses and other GIS data were used to perform a network analysis for the optimal routes for warfarin monitoring visits. These measures of revealed accessibility were then used in the cost model to measure monetary time and travel costs in addition to direct healthcare costs of anticoagulation management.

**Results:**

The share of time and travel costs in warfarin monitoring is 26.6% of the total therapy costs in our study region. With current drug retail prices in Finland, the societal expense of anticoagulation management is only 2.6% higher with DOACs than in the baseline with warfarin. However, when 25% lower distributor’s prices are used, the total societal cost decreases by 13.6% with DOACs.

**Conclusions:**

Our results indicate that patients’ time and travel costs critically increase the societal cost of warfarin therapy; and despite the higher price of DOACs, they are already cost-efficient alternatives to warfarin in anticoagulation management. In the future, the cost of AF complications should be included in the cost comparison between warfarin and DOACs. Our modeling approach applies to different geographical regions and to different healthcare processes requiring patient monitoring.

## Background

Atrial fibrillation (AF), which is associated with increased risk of ischemic stroke (IS), systemic embolism, heart failure, and mortality [1], is the most common arrhythmic condition in developed countries. With increasing prevalence, especially among population over 65 years old, it has become a significant public health problem and a cause of increasing healthcare expenditure. Previously, warfarin has been the primary recommended anticoagulant for reducing the risk of IS. But the current recommendation given by the European Society of Cardiology also emphasizes the benefits of direct oral anticoagulants (DOACs) [2].

As warfarin has a narrow therapeutic range, a safe use of it requires regular monitoring of the anticoagulation effect through blood tests. The international normalized ratio (INR) target range measured by a blood test is set between 2 and 3 [3]. Underanticoagulation may lead to thrombotic events, and overanticoagulation has the risk of hemorrhage [3, 4]. Yet the monitoring can be a significant burden for patients as it involves a number of clinic or laboratory visits dependent on the achieved INR level [5]. Compared with warfarin, direct oral anticoagulants (DOAC) seem to have some therapeutic benefits such as the reduction of hemorrhagic strokes and systemic embolic events [6], and patients with DOACs do not require regular laboratory monitoring. On the other hand, the higher price of DOACs has maybe thus far been reducing their use in anticoagulation management.

The cost-effectiveness of DOACs has been assessed in multiple studies [7–9]. Respectively, patients’ travel costs associated with the warfarin therapy have been addressed in a few studies [10–13] . However, to our knowledge, only one study has considered time and travel costs when comparing the total costs of anticoagulation management for warfarin and DOACs. Marcolino et al. [14] report that in the Brazilian context, the cumulative costs per patient using warfarin with follow-up in anticoagulation clinics is currently higher than the strategy of using DOACs. This outcome is not surprising, but this topic could also be studied further, as the patient data for the study of Marcolino et al. [14] was collected from a single anticoagulation clinic using a time period of just 3 months.

The opportunity costs of medical choices require further investigation also in the geographical context, as the shift from warfarin to DOACs would eliminate the burden of INR monitoring and create societal savings when time and travel costs are considered. Additionally, the increment of DOAC therapy would mitigate the importance of access to healthcare in anticoagulation management especially in sparsely populated rural areas where the health services are harder and more expensive to reach due to longer distances. For regularly monitored medical conditions, travel time and distance can even create barriers to effective use of services [15, 16].

Traveling needed to reach health services incurs costs to both patients and society; but all additional costs, including travel costs and the value of lost leisure time and production loss, are often ignored in economic evaluations, which tend to focus on the direct monetary costs of care [10, 11]. Nevertheless, it has been shown that patient time and travel costs associated with receiving healthcare services may be critical, and they should be included in the economic assessments of interventions that require regular monitoring and traveling [10, 17–19]. Considering the total cost of an intervention, patient time and travel costs have been reported to be 21% in type 2 diabetes follow-up in Finland [17] and 20–40% in cancer screening in the UK [18].

### Aim of the study

With the higher price of DOAC drugs but minuscule monitoring costs compared with warfarin, it is important to measure and compare the total costs of these two alternative anticoagulation managements. Thus far, the previous cost comparisons including time and travel costs have not covered larger geographical areas or used electronic health records (EHRs). Hence, the aim of our study was to use patient register data to measure revealed healthcare accessibility as monetary cost, and to investigate the potential savings of travel and time costs achievable with the shift from warfarin to DOACs. We evaluated whether these simulated savings are enough to create societal savings in the total costs of anticoagulation management in a regional public healthcare setting in North Karelia Eastern Finland.

## Methods

### Study region and patient data

The study area in Eastern Finland includes the region of North Karelia and the nearby municipality of Heinävesi, which belongs to the same healthcare district of Siun sote (14 municipalities, 166,000 inhabitants, a population density of 8.8 per km^2^ (22.9 per mi^2^), degree of urbanization 76.2%). The population is distributed unevenly among more densely populated centers and sparsely populated rural areas. Specialized healthcare services can be acquired from the central hospital and primary care services from 23 public healthcare centers.

The unique characteristic in the region in Finland is the common electronic patient database used by all municipalities. This regional patient database (Mediatri) holds all public healthcare records from the healthcare district. For this study, all AF (ICD-10 code I48) patients (*N* = 6519) having the diagnosis day between 1.1.1996 and 12.31.2016 were included in the study with the conditions that they were alive at the end of 2017, they had at least one healthcare visit with an AF diagnosis between 2014 and 2017, and their home address could be geocoded. The measurement timeline for this patient sample was the year 2017, for which the data retrieval from Mediatri consisted of individual-level patient data, such as gender, age, domicile, diagnoses, laboratory results, prescriptions and healthcare center visits.

As we were interested in the medication use in 2017, we identified patients who had recently switched from warfarin to a DOAC medication and assumed that DOAC prescription, even overlapping with warfarin prescription, at the end of the year 2017 indicated that the patient had switched to DOAC during the year. These patients were counted as DOAC users in addition to patients who had been using DOAC for more than a year. The rest of the patients with warfarin prescription but without DOAC prescription were treated as warfarin users, if they had at least 1 INR measurement in 2017. This additional criterion helped to confirm the warfarin use, as our data lacked the information whether the patients have redeemed their warfarin prescriptions. However, following this decision all patients who might have be using warfarin without going to INR monitoring were excluded from the group of warfarin users.

In Finland, INR is routinely measured at sample collection points at local clinics (called an INR sampling point in this study). The results from both normal laboratory measurements and from INR quick tests performed by nurses are registered in the same database. After a laboratory measurement, the patient receives instructions for warfarin dosage adjustment by an SMS message or in some cases, by a phone call. For warfarin users in North Karelia, the average distance to the closest INR sampling point along a road network is 6.2 km. We checked the number of patients using self-monitoring devices in the region, but due to the low number of users (*N* = 23), self-monitoring was eventually not considered in the study setting.

### The cost model

We measured both the patients’ costs of travel and time loss and direct anticoagulation management costs using a georeferenced cost model, which is an application of the previous model for the travel and time costs of type 2 diabetes by Leminen et al. [17]. The model was developed further in order to measure the societal costs of anticoagulation management performed with either warfarin or DOACs. The model consists of patient travel costs with four different travel modes based on a network analysis, the monetary value of patient time loss associated with traveling and INR measurements, and direct anticoagulation management costs (such as the cost of INR blood tests and the medication costs of warfarin or DOACs).

These costs can be expressed with equations for every travel mode, similarly to the previous studies of Ford et al. [20] and Leminen et al. [17]. Walking (*C*_*WALK*_), private car (*C*_*CAR*_), bus (*C*_*BUS*_) and taxi (*C*_*TAXI*_) are expressed with the following equations:

1a $$ {C}_{WALK}=T\ast VOT\ast P $$

1b $$ {C}_{CAR}=\left(T+{T}_p\right)\ast VOT\ast P+D\ast VOC $$

1c $$ {C}_{BUS}=\left(T+{T}_a\ \right)\ast VOT\ast P+F $$

1d $$ {C}_{TAXI}=\left(T+{T}_a\ \right)\ast VOT\ast P+F+D\ast VOC $$

where *T* is the travel time, *VOT* (value of time) is the gross wage coefficient of the patient’s zip code area, *P* is the patient’s productivity coefficient (used as weight for *VOT* to depict patient’s lost contribution to the society based on lost working time and leisure), *T*_*p*_ is the vehicle parking time, *D* is the road distance in km, *VOC* is the vehicle operating cost per km, *T*_*a*_ is the access time to the network (walking time to a bus stop or from a bus stop to the clinic or laboratory, waiting time at the bus stop, or service time in a taxi), and *F* is the bus fare or the fixed charge of taxi paid for the journey. Anticoagulation management related costs are calculated differently for warfarin therapy (*C*_*WARF*_) and DOAC therapy (*C*_*DOAC*_):

2a $$ {C}_{WARF}={T}_{inr}\ast VOT\ast P+{C}_{inr}+{C}_t+{C}_m $$

2b $$ {C}_{DOAC}={C}_m $$

where *T*_*inr*_ is the time spent in the INR monitoring visit and adjusting the warfarin dosage according to counselling via SMS, *VOT* (value of time) is the gross wage coefficient of the patient’s zip code area, *P* is the patient’s productivity coefficient (used as weight for *VOT* to depict patient’s lost contribution to the society based on lost working time and leisure), *C*_*inr*_ is the cost of the INR monitoring visit, *C*_*t*_ is the cost of the INR blood test (sampling and blood test), and *C*_*m*_ is the cost of medication (warfarin or DOACs).

**Fig. 1:**
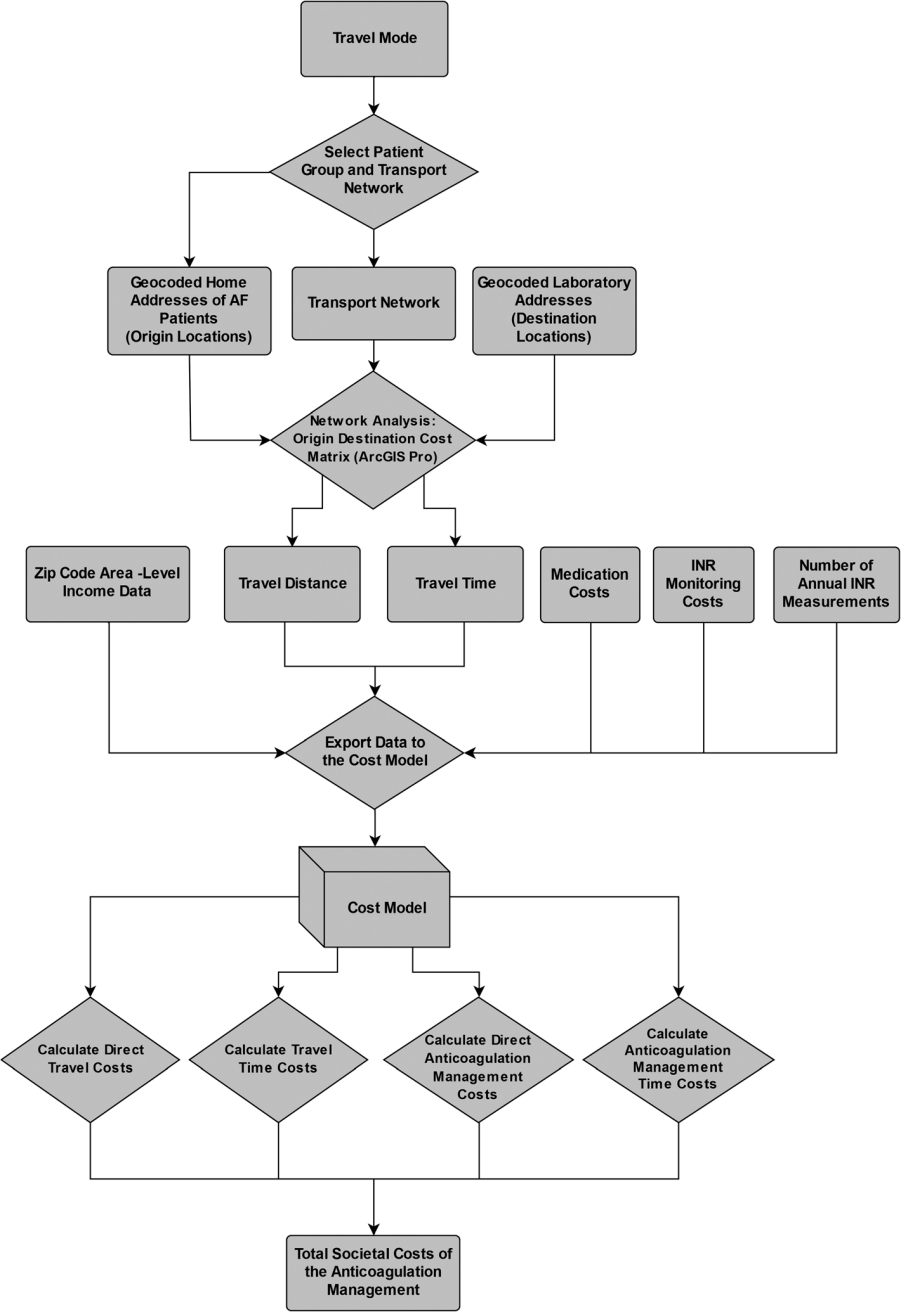
Flow chart of the cost model

The cost model was executed following the flow chart presented in Fig. 1. At baseline, the costs were calculated based on the medication data from 2017. Next, we designed a scenario where warfarin was replaced with DOACs for patients who had no contraindications for the drug. Thus, because of safety concerns or lack of sufficient evidence, patients with a prosthetic heart valve or chronic kidney disease (*N* = 296) were determined to continue using warfarin.

Additionally, warfarin users with less than 5 annual INR measurements (*N* = 553) were excluded from the new DOAC users, as we had evidence of a measurement registration problem in the area of 3 municipalities. These excluded patients had stayed long periods in nursing homes and wards, and despite close monitoring, most of them had only few registered INR measurements in 2017. Additional measurements made by nurses were not registered in the patient database due to the differences in medical practice. Thus, based on the registered data, these patients did not represent regularly monitored patients and the switch to DOAC would have increased costs unrealistically for them. It should be noted, though, that this leaves the possibility of a small number of patients being excluded, even though their low number of INR measurements might be correct, and they just did not go for their appointed measurements.

### Travel modes

When measuring travel time and travel costs, the choice of travel mode is the first thing to identify. The determination of each patient’s travel mode can be a challenge, as the travel mode choice is influenced by multiple factors such as age, gender, income, education, employment, family size, number of children, and car ownership [21–23]. Thus, with limited data and without time-consuming inquiries, some generalizations are necessary. A high age associated with diseases like atrial fibrillation changes patients’ travel patterns, especially if they are entitled to travel cost reimbursements, like in Finland. Travel expenditures are usually compensated for according to the least expensive travel mode, but more expensive taxi trips are accepted for health reasons or when suitable public transport is not available.

Four travel modes—private car, taxi, walking and bus—for patients using warfarin were selected following the criteria in Table 1. These criteria are based on the classifications made in the previous study by Leminen et al. [17] in the same study area. From a total of 4560 patients using warfarin, 3961 were included in the travel cost analysis. Additional 599 warfarin users in sheltered homes were excluded, but the cost of annual INR measurements was still calculated for this patient group.

**Table 1:** Characteristics of selected travel modes

Travel mode	Number of patients	Criteria	Travel speed
Private car	2132	Distance to the INR sampling point > 1 km, bus not an option and patient age < 80 years or distance to the INR sampling point > 0.2 km and patient age < 85 years	Road speed limit
Taxi	925	Patient age ≥ 85 years	Road speed limit
Walking	546	Distance to the closest INR sampling point ≤1 km and patient age < 80 years, or distance ≤0.2 km and patient age < 85 years	4 km/h
Bus	358	Distance to the closest INR sampling point > 1 km, destination accessible by bus, distance to the closest bus stop ≤0.25 km and patient age < 80 years	30 km/h (average speed based on timetables)
Total	3961		

### GIS based network analysis

From a patient’s perspective, the effort and cost to reach an anticoagulation clinic or laboratory are dependent on the accessibility of the services used. The geographical accessibility and availability of services, commonly called spatial accessibility [24–26], is affected by the locations of destinations (supply) and starting points (demand) as well as the performance of the transportation system [27].

Accessibility to healthcare services is usually measured by distance, travel time, or monetary costs; and for large areas, this can be done most easily with GIS (geographic information system) methods using either vector- or raster-based analysis [28]. The use of vector-based network analysis has increased along with the better availability of transport network high-quality data [29]. New measures such as the shortest and fastest routes based on the road network also yield more accurate results compared with a simple straight-line distance [30]. The measured accessibility can be both potential (when focusing on the hypothetical use of the available healthcare services) and revealed (when measuring the actual utilization of the resources) [24, 26, 31]. Our study setting builds on the revealed accessibility, as the trip frequency is based on patient information and the real number of INR monitoring visits in 2017.

We conducted the GIS based network analysis using the *Origin-Destination (OD) Cost Matrix* method [32] in Esri ArcGIS Pro 2.2 software (Esri, Redlands, CA, USA). The used road network data was modified from the Digiroad database by the Finnish Transport Agency. Optimal routes between patient home addresses and INR sampling points were calculated based on travel time, as previously suggested by Ray & Ebener [28] and the World Health Organization (WHO) [33]. Additionally, travel distance was saved simultaneously based on these fastest routes. Because the study area has no notable traffic congestion and the INR monitoring is premeditated, the rush hour variability of accessibility was not needed in travel times. The calculated travel time and travel distance for two-way journeys were later converted as monetary costs in the cost model (Fig. 1).

### Cost values and sensitivity analysis

The cost of travel, the time cost of both travel and therapy, and the clinical cost of INR measurements and monitoring were calculated using parameters in Table 2 for eqs. (1a)–(2a). The time spent on traveling and INR monitoring was valued based on the average hourly income derived from the patient’s zip code area income data of 2017. Additionally, following Jowett et al. [10], a separate coefficient was used to weigh the productivity level of working-age (< 63 years) and retired (≥ 63 years) patients. The time loss for a working-age patient was considered equal to the average hourly gross wage, and the leisure time for a pensioner was valued at 35% of the average wage. All other monetary values, including the cost of medication, were based on the current prices (October 1, 2018) in the study area.

**Table 2:** Parameters of the model

Parameter	Description	Value	Unit	Additional information
*T*	Travel time using the fastest route on a road network		min	Calculated with network analysis
*VOT*	The value of time based on the average hourly income of a zip code area		EUR	Average hourly gross wage converted from the monthly gross wage in 2017
*P*	Patient’s productivity coefficient	1.00 or 0.35	Used as weight	Depicts patient’s lost contribution to the society. Working time is valued as 100% and leisure time as 35% of the hourly wage
*T*_*p*_	Private car parking time	5	min	Added to the total journey time
*D*	Travel distance calculated from the fastest route on a road network		km	Calculated with network analysis
*VOC*	Vehicle operating cost			
	Private car	0.45	EUR/km	Includes fuel cost and vehicle maintenance and depreciation costs
	Taxi	1.60	EUR/km	Most common fare per km
*T*_*a*_	Network access time			
	Taxi service time	5	min	Added to the total journey time
	Waiting time in a bus stop	7	min	Added to the total journey time
	Walking time to a bus stop	5	min	Added to the total journey time
	Walking time from a bus stop to home or clinic	5	min	Added to the total journey time
*F*	Fare paid for the journey			
	One-way bus fare	2.00, 3.80 or 5.00	EUR	Fare depends on the fare zone
	Taxi initial fixed charge	5.90	EUR	Most common fee in the study area
*T*_*inr*_	Patient time loss associated with INR monitoring	40	min	Blood test and the adjustment of warfarin dosage
*C*_*inr*_	Cost of the INR monitoring visit	25.00	EUR	Includes healthcare personnel costs (result examination, warfarin dosage counseling via SMS or phone call and making the next appointment) based on the service provider prices for nurse/doctor phone consultation
*C*_*t*_	Cost of the INR blood test	10.50	EUR	Sampling 7.50 € + test 3.00 €
*C*_*m*_	Cost of the medication			
	Warfarin	3.90/2.20	EUR/mo.	Retail price (excl. VAT)/distributor’s price. Calculated with the average consumption of 5 mg/day
	Apixaban	82.40/59.50	EUR/mo.	Retail price (excl. VAT)/distributor’s price
	Dabigatran	82.40/59.50	EUR/mo.	Retail price (excl. VAT)/distributor’s price
	Rivaroxaban	74.00/59.40	EUR/mo.	Retail price (excl. VAT)/distributor’s price
	Edoxaban	73.80/59.40	EUR/mo.	Retail price (excl. VAT)/distributor’s price

All monetary values, except the value of time, are based on the prices (EUR) on October 1, 2018

For the costs of warfarin and DOAC medications, we used national retail prices (excluding VAT), as well as lower drug distributor’s prices, which can be regarded as the wholesale prices. The retail prices reflected the real value, which included the statutory profit margin of Finnish pharmacies. The alternative distributor’s prices were vital, as our measurement goal was to measure the social opportunity costs of the two medical choices, and unlike for warfarin, the price for DOACs was crucial in this regard. These wholesale prices without any added value offer useful information for the decision-makers about the economic viability of the transitioning from warfarin to DOACs. In the scenario, both prices were presented as the average price of four different DOACs.

As travel costs depend on the distance and the number of trips, the sensitivity of the travel costs was simulated for the artificial travel distance (2 km, 5 km, 10 km, 20 km, and 30 km) and for the artificial number of annual INR monitoring visits (5, 10, 15, and 20), with the assumption that each trip is made by private car. Additional sensitivity analysis for the costs of warfarin therapy included the artificial number of annual INR measurements (5, 10, 15, and 20) and the different values for leisure time (20, 35 and 50% of the average gross wage per hour) due to the lack of profound consensus on its valuation. Here we wanted to test the impact of different values on time costs by adding two arbitrary values around the value of 35% suggested earlier by Jowett et al. [10].

## Results

### Characteristics of the patient group

Approximately 4.0% (*N* = 6594) of the population living in the healthcare district had a diagnosed AF at the end of 2016. After geocoding the home addresses of this patient sample with a success rate of 98.9%, the final number of patients included in the study was 6519. The mean age of these patients was 76.5 years, and 54.2% were men. Of this patient group, 94.0% had at least one INR measurement between 2014 and 2017, and the time in therapeutic range (TTR) for 2017 could be determined for 72.5% of the patients with warfarin. The detailed characteristics for the geocoded patient group are shown in Table 3.

**Table 3:** Characteristics of the patient group

Variable	All geocoded patients (*N* = 6519)	Warfarin (*N* = 4560)	DOACs (*N* = 1239)	No medication (*N* = 720)
Age, mean (SD)	76.5 (10.5)	78.1 (9.4)	75.1 (10.1)	68.5 (13.5)
Retired, age ≥ 63 years, n (%)	5896 (90.5)	4302 (94.3)	1103 (89.0)	491 (68.2)
Gender, male, n (%)	3532 (54.2)	2388 (52.4)	663 (53.5)	481 (66.8)
BMI, mean (SD)	29.1 (6.2)	29.3 (6.4)	29.3 (5.8)	28.1 (5.4)
Obesity (BMI > 30), n (%)	1456 (22.3)	1006 (22.1)	327 (26.4)	123 (17.1)
CHA2DS2-VASc, mean (SD)^a^	3.1 (1.6)	3.3 (1.5)	3.1 (1.6)	1.9 (1.6)
Diabetes, n (%)	1648 (25.3)	1210 (26.5)	315 (25.4)	123 (17.1)
Hypertension, n (%)	3261 (50.0)	2302 (50.5)	696 (56.2)	263 (36.5)
Vascular disease, n (%)^b^	1657 (25.4)	1219 (26.7)	323 (26.1)	115 (16.0)
Congestive heart failure, n (%)	976 (15.0)	771 (16.9)	161 (13.0)	44 (6.1)
Transient ischemic attack (TIA), n (%)	271 (4.2)	188 (4.1)	73 (5.9)	10 (1.4)
Home address in assisted living building, n (%)	765 (11.7)	599 (13.1)	93 (7.5)	73 (10.1)
Number of INR measurements in 2017, mean (SD)	15.4 (11.0)	15.9 (10.9)		
Standard TTR definable in 2017, n (%)	3524 (54.1)	3307 (72.5)		

^a^Anticoagulation medication should be used for patients having the score of 2 or more

^b^Vascular disease includes the following ICD-10 codes: I20-I25, I70.9

**Fig. 2:**
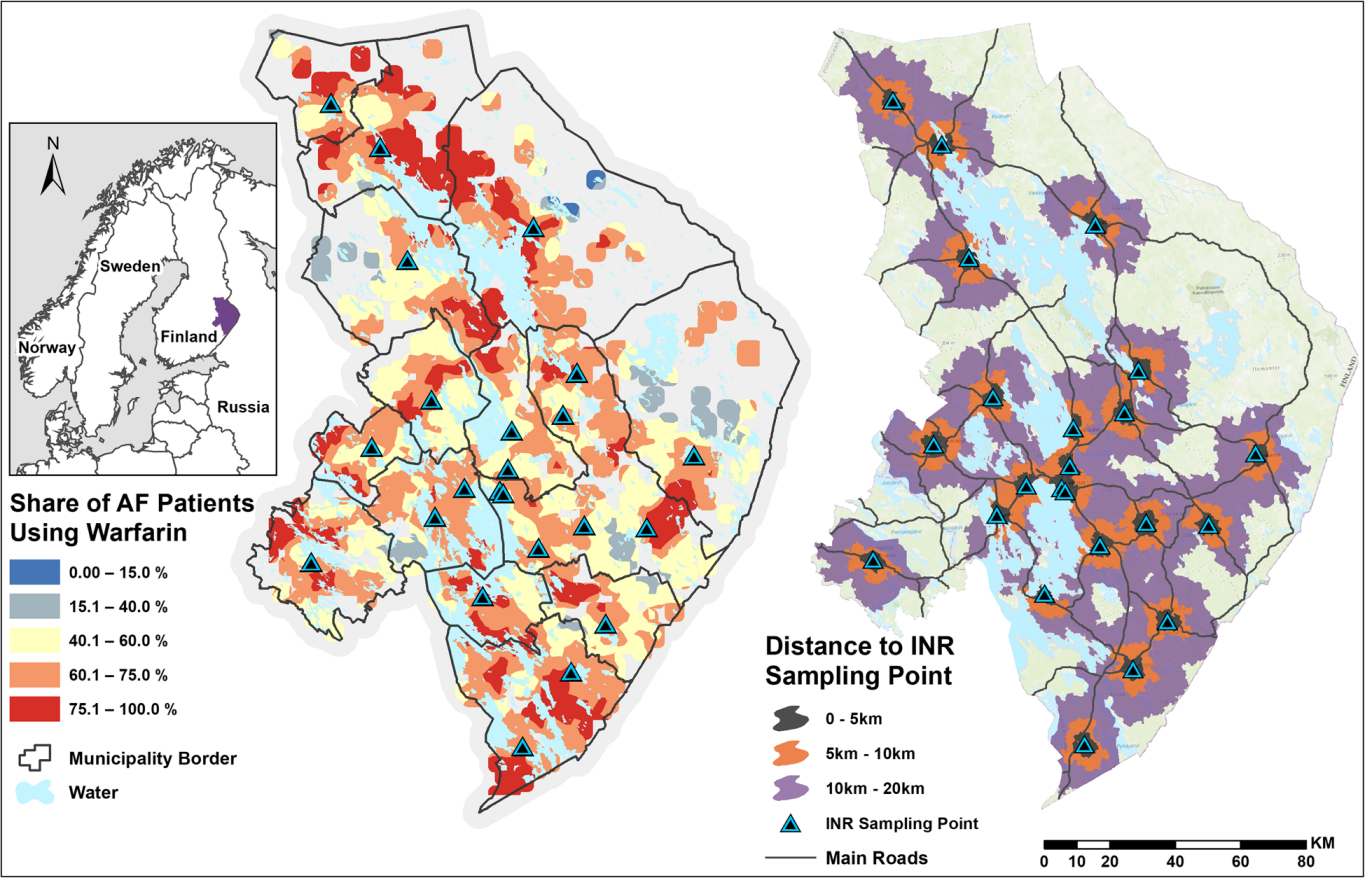
Geographical differences in the usage of warfarin, and the travel distances to INR sampling points along the road network. The map on the left is based on Empirical Bayesian Kriging (EBK) interpolation for patient locations in a 2 km × 2 km grid. The figure has been generated with ArcGIS 10.5 software (Esri, Redlands, CA, USA), and it is freely available to use

When classified by the used medication, 70.0% (*N* = 4560) of patients had a warfarin prescription and at least 1 INR measurement in 2017. Respectively, 19.0% (*N* = 1239) of the patients had a DOAC prescription (6.4% apixaban, 6.4% rivaroxaban, 6.1% dabigatran, and 0.1% edobaxan). The share of the patients without medication was 11.0% (*N* = 720). Surprisingly, the usage of warfarin was most common in distant areas, where the travel distance to an INR sampling point is long (Fig. 2).

### Cost analysis

In our study area, warfarin therapy costs for the patient group were approximately 3,800,000 EUR / 4,410,000 USD (1 EUR = 1.1606 USD, on October 1, 2018) in 2017 when considering both direct costs and the cost types regarded as indirect costs (Table 4). Overall, these indirect costs constitute 26.6% of the total annual costs.

**Table 4:** Baseline: Annual costs of warfarin therapy (2018 prices)

	Annual Cost (EUR)	Share (%)	Per Patient (EUR)	
Total cost of warfarin therapy:	3,789,930	100.0		
Direct costs	2,781,820	73.4	610	(*N* = 4560)
INR measurements and monitoring	2,570,450	67.8	564	(*N* = 4560)
Warfarin medication	211,370	5.6	46	(*N* = 4560)
Indirect costs	1,008,110	26.6		
Time costs of INR measurements and monitoring	193,020	5.1	42	(*N* = 4560)
Travel costs	715,990	18.9	181	(*N* = 3961)
Travel time costs	99,100	2.6	25	(*N* = 3961)

The annual travel costs in warfarin therapy in the study area are 815,090 EUR / 945,990 USD (1 EUR = 1.1606 USD), which is an average of 206 EUR / 239 USD per patient and 14 EUR / 16 USD per journey (Table 5). Of the travel costs, 87.8% are direct costs and 12.2% are time costs. In the cost model, private car and taxi are the most used travel modes, and this is also displayed in their large share of the total travel costs. Taxi is the most expensive mode of travel even in short distances. Thus, pensioners have on average higher travel costs than working-age patients, as patients 85 years old and above were expected to use a taxi. However, because of the lower valuation for leisure time, the travel costs for pensioners are relatively lower by private car, bus, and walking.

**Table 5:** Baseline: Travel costs in warfarin therapy (2018 prices)

Costs (EUR)	Private Car (*N* = 2132)	Taxi (*N* = 925)	Bus (*N* = 358)	Walking (*N* = 546)	All Travel Modes (*N* = 3961)
Total annual cost	285,790	464,850	53,200	11,250	815,090
Direct travel costs	235,260	444,360	36,370		715,990
Time costs	50,530	20,490	16,830	11,250	99,100
Average annual cost per patient	134	503	149	21	206
Average monthly cost per patient	11	42	13	2	17
Average cost of one journey	9	29	11	2	14
Patient age	Average annual cost (the cost of one journey in parentheses)				
Age < 63 years	190 (15)		191 (16)	52 (4)	170 (13)
Age ≥ 63 years	129 (9)	512 (29)	141 (10)	18 (1)	208 (14)
Travel distance	Average annual cost (the cost of one journey in parentheses)				
Distance < 5 km	42 (3)	310 (18)	133 (10)	21 (2)	117 (8)
Distance 5–10 km	121 (8)	701 (38)	167 (12)		220 (15)
Distance 10–20 km	229 (16)	870 (59)			362 (25)
Distance ≥20 km	395 (29)	2006 (102)			662 (45)
INR measurements	Average annual cost (the cost of one journey in parentheses)				
INR M. per year ≤6	33 (11)	124 (36)	32 (11)	4 (1)	44 (14)
INR M. per year 7–12	91 (9)	277 (28)	105 (11)	15 (2)	117 (12)
INR M. per year 13–20	152 (10)	454 (28)	172 (11)	23 (1)	214 (13)
INR M. per year > 20	246 (8)	912 (30)	324 (11)	46 (2)	427 (14)

We also performed two sensitivity analyses. First, the sensitivity of the costs of warfarin therapy was tested using three different valuations of leisure time and four numbers of INR monitoring visits. The results in Table 6 show that the valuation of leisure time has a minor effect on the total costs of warfarin therapy. With each number of annual INR measurements, the share of indirect costs is approximately 2 percentage points higher when leisure time is valued at 35% of the average gross wage per hour instead of 20%, or at 50% instead of 35%. Depending on the number of measurements and the value of lost leisure time, the share of indirect costs varies between 21.9 and 29.0% compared to 26.6% in the baseline.

**Table 6:** Sensitivity analysis for the costs of warfarin therapy (2018 prices)

Annual cost EUR (share % from the total cost)	Number of annual INR measurements:			Number of annual INR measurements:			Number of annual INR measurements:			Number of annual INR measurements:		
	5			10			15			20		
	The value of leisure time (%/gross wage per hour):			The value of leisure time (%/gross wage per hour):			The value of leisure time (%/gross wage per hour):			The value of leisure time (%/gross wage per hour):		
	20	35	50	20	35	50	20	35	50	20	35	50
Total cost of warfarin therapy:	1,291,770 (100)	1,324,700 (100)	1,357,630 (100)	2,372,160 (100)	2,438,030 (100)	2,503,890 (100)	3,452,560 (100)	3,551,360 (100)	3,650,150 (100)	4,532,950 (100)	4,664,690 (100)	4,796,410 (100)
Direct costs	1,009,370 (78.1)	1,009,370 (76.2)	1,009,370 (74.3)	1,807,370 (76.2)	1,807,370 (74.1)	1,807,370 (72.2)	2,605,370 (75.5)	2,605,370 (73.4)	2,605,370 (71.4)	3,403,370 (75.1)	3,403,370 (73.0)	3,403,370 (71.0)
INR measurements and monitoring	798,000	798,000	798,000	1,596,000	1,596,000	1,596,000	2,394,000	2,394,000	2,394,000	3,192,000	3,192,000	3,192,000
Warfarin medication	211,370	211,370	211,370	211,370	211,370	211,370	211,370	211,370	211,370	211,370	211,370	211,370
Indirect costs	282,400 (21.9)	315,330 (23.8)	348,260 (25.7)	564,790 (23.8)	630,660 (25.9)	696,520 (27.8)	847,190 (24.5)	945,990 (26.6)	1,044,780 (28.6)	1,129,580 (24.9)	1,261,320 (27.0)	1,393,040 (29.0)
Time costs of INR measurements and monitoring	35,640	57,040	78,440	71,270	114,080	156,880	106,910	171,120	235,320	142,550	228,160	313,770
Travel costs	224,700	224,700	224,700	449,410	449,410	449,410	674,110	674,110	674,110	898,810	898,810	898,810
Travel time costs	22,060	33,590	45,120	44,110	67,170	90,230	66,170	100,760	135,350	88,220	134,350	180,460

The second sensitivity analysis was done for the annual travel costs (Table 7) using the five distances and the four numbers of INR monitoring visits. As the distance would not have been suitable measure for all four travel modes, for these sensitivity results every patient was determined to use private car. Reportedly, costs increase linearly with increasing distance and the increasing number of trips.

**Table 7:** Sensitivity analysis of the travel costs when everybody travels by car (2018 prices)

Distance (km) by private car	Annual travel costs (EUR)				Cost per journey (EUR)
	Number of annual INR monitoring visits:				
	5	10	15	20	
2	53,860	107,720	161,580	215,450	2.7
5	114,320	228,650	342,970	457,290	5.8
10	215,080	430,150	645,230	860,310	10.9
20	416,600	833,200	1,249,800	1,666,400	21.0
30	618,110	1,236,220	1,854,330	2,472,430	31.2

Finally, we estimated the cost change of the shift of all eligible patients from warfarin to DOAC (Table 8). In the tested scenario, 81.4% (*N* = 3711) of the patients currently using warfarin were shifted to use DOACs, and 18.6% (*N* = 849) were determined to continue using warfarin due to the formerly described restrictions to DOACs or due to a falsely low number of annual INR measurements. With retail prices excluding VAT, the total cost of anticoagulation management would increase 2.6% when warfarin is replaced with DOAC for as many patients as possible. In the alternative DOAC scenario, applying the drug distributor’s pricing, the total cost decreases 13.6%.

**Table 8:** Cost change in the scenario: shift from warfarin to DOAC (2018 prices)

Annual costs in EUR (share % from total costs)	Drug retail prices (excl. VAT)			Distributor’s drug prices		
	Baseline	Scenario	Change	Baseline	Scenario	Change
Costs of INR monitoring:	2,974,840 (59.8)	343,500 (6.7)	−88.5%	2,881,800 (62.9)	326,180 (8.2)	−88.7%
INR measurements and medication (*N* = 4560)	2,781,820 (56.0)			2,688,780 (58.7)		
Time costs of INR monitoring (*N* = 4560)	193,020 (3.8)			193,020 (4.2)		
Patients unable to switch to DOAC (*N* = 296)		270,230 (5.3)			264,190 (6.7)	
Patients not switching to DOAC (< 5 INR M. 2017, *N* = 553)		73,270 (1.4)			61,990 (1.5)	
Travel costs:	815,090 (16.4)	97,840 (1.9)	−88.0%	815,090 (17.8)	97,840 (2.5)	−88.0%
Direct travel costs (*N* = 3961)	715,990 (14.4)			715,990 (15.6)		
Travel time costs (*N* = 3961)	99,100 (2.0)			99,100 (2.2)		
Patients continuing warfarin (*N* = 772)		97,840 (1.9)			97,840 (2.5)	
Cost of DOAC therapy:	1,181,850 (23.8)	4,661,180 (91.4)	294.4%	883,970 (19.3)	3,531,580 (89.3)	299.5%
Patients using DOAC (*N* = 1239)	1,181,850 (23.8)	1,181,850 (23.2)		883,970 (19.3)	883,970 (22.3)	
Patients switching from warfarin to DOAC (*N* = 3711)		3,479,330 (68.2)			2,647,610 (67.0)	
Total cost of anticoagulation management:	4,971,780 (100)	5,102,520 (100)	2.6%	4,580,860 (100)	3,955,600 (100)	−13.6%

## Discussion

The hindrance to the extensive shift from warfarin to DOACs in anticoagulation management has been the high price of the new drugs. However, when measuring the total societal costs of the therapy, the indirect costs of warfarin are often ignored. Our modeled results in the region of North Karelia show that when INR is measured routinely in local clinics, travel and time costs can constitute over 25% of the total societal costs of warfarin therapy.

In our study area, the mean travel cost per INR monitoring visit (including time costs) varies from 2 to 29 EUR depending on the travel mode, with an average of 13.5 EUR for all travel modes. A previous multinational study by Jowett et al. [10] conducted by questionnaire reported mean patient costs (including travel costs, fee paid by patients, and the time costs of travel and clinic attendance) per visit of 12.8 EUR in Australia, 19.5 EUR in Spain, 18.3 EUR in Sweden, and 15.6 EUR in the UK (adjusted for inflation from 2003 to 2018). To fully compare our results with these previous results, the average time cost of INR measurement and warfarin dosage adjustment (2.7 EUR) must be added to the travel related costs. After this adjustment, the comparable mean patient cost per visit is 16.2 EUR in our study area. This is well in line with the findings by Jowett et al., considering that these two studies were executed with different methods. It is also a decent indication that modeling can be used to achieve comparable travel cost results with questionnaires, especially when dealing with large-scale patient groups and areas.

The sensitivity analysis for the travel costs of INR monitoring shows that the costs increase in line with travel distance and the number of monitoring visits. Respectively, the total cost of warfarin therapy is also highly dependent on the number of annual measurements. Additionally, the sensitivity analysis for the valuation of leisure time suggests that the value of time has a moderate effect on the share of indirect costs in warfarin therapy.

Our investigation on the maximal societal savings achievable with the shift from warfarin to DOACs shows that the total costs with these two forms of therapy can be very similar and comparable or very different, depending on the price of the DOACs. Hence, if this study is applied in different countries, the results vary between regions. With current Finnish retail prices (excluding VAT), the transition to DOAC therapy would increase the societal costs by 2.6% compared to baseline, based on the current patient group in our study area. Respectively, with lower distributor’s prices, the costs would decrease 13.6% (in total 625,000 EUR / 725,000 USD, 1 EUR = 1.1606 USD). From a societal perspective, in our study area this means that when considering the time and travel costs in INR monitoring, DOAC therapy is currently cost-efficient but not cheaper than warfarin therapy. However, presuming that drug prices will decrease in the future as the expiration of most DOAC patents by 2023 allows the introduction of first generics, the savings could be considerable when preferring DOAC therapy over warfarin.

Marcolino et al. [14] reported that in the Brazilian context, the cost of anticoagulation management with DOACs is lower than with warfarin. As the average monthly price for DOACs in this study was even lower than the distributor’s prices in our study (54 USD vs. 68 USD, adjusted for inflation and converted on October 1, 2018), both leading to savings when compared with the total costs of warfarin therapy, these drug prices can be used as raw estimates for the level at which societal savings are currently achievable.

The shift from warfarin to DOACs not only has an impact on the societal cost of anticoagulation management, it also removes the burden of INR monitoring and traveling. This decreases the importance of the spatial accessibility of health services, contributing to better geographical equality of anticoagulation management and healthcare in general. The shift would be even more rational from a patient’s perspective, as in Finland after the drug reimbursement by Social Insurance Institution, the purchase price of DOACs for patients is only 35% of the original retail price. However, this out-of-pocket expense was intentionally overlooked in this study, as we wanted to investigate the societal lucrativeness and economic viability of the replacement of warfarin by DOACs.

Our study reviews the accessibility setting and travel costs in a single healthcare district in Finland. These results are most relevant in sparsely populated rural regions (population density of 5–20 per km^2^ / 15–50 per mi^2^, degree of urbanization lower than 75%) with a high proportion of elderly population (median age of the population over 45 years). In Europe, comparable regions can be found, for example, from other parts of Finland and Scandinavia [34]. Many US counties also meet these characteristics [35, 36]. Within a healthcare district, the shift from warfarin to more expensive DOACs is less cost-efficient in urban areas where the population has better access to healthcare, and the sample collection points for INR monitoring are on average closer than in our study region. As DOAC therapy is less often the cheaper option in urban areas, in many cases the societal costs might be lower when using warfarin.

Our modeling setting has some limitations. Firstly, the effectiveness of both interventions was assumed to be equivalent. Yet DOACs have been shown to offer a better safety profile, as they possess a lower risk of fatal and costly complications (such as ischemic strokes or major bleeding) associated with warfarin. The cost of those complications was beyond the scope of this study, which means that, as indicated previously [37, 38], the cost reduction enabled by DOACs is most likely even greater than in our study setting. Secondly, the time loss of a possible escort for INR monitoring visits made by older patients was not considered, which for its part leads to a slight underestimation of the total societal cost of INR monitoring in warfarin therapy. Thirdly, we left the temporal variability of accessibility unconsidered, even though it might affect the availability of services in some frequently visited laboratories.

The research use EHRs is increasing, as they enable large-scale, up-to-date studies [39]. By combining patient register data with GIS methods, health research can be spatialized. This opens new possibilities for the assessment of regional health differences, and it provides real-world information for healthcare planning. Our modeling approach can be used as a baseline for measuring time and travel costs of different healthcare processes that require patient monitoring. The model can also be expanded and developed further to suit different geographical regions with alternative travel patterns.

## Conclusion

As a conclusion from our study setting, the results suggest that the amount of patients’ travel and time costs critically increase the societal cost of INR monitoring and warfarin therapy, and these cost types should not be overlooked. From a societal perspective, despite the higher price of DOAC drugs, they are a cost-efficient alternative to warfarin in anticoagulation management. As a more comprehensive continuation in the future, it would be important to also include the cost of AF complications in the cost comparison between warfarin and DOACs. In addition to the costs modeled in this study, also the effectiveness of warfarin and DOACs should be assessed using the same patient sample.

## Data Availability

The datasets generated and/or analyzed in this study are not publicly available due to the confidentiality of individual-level patient data.

## Abbreviations

AF: Atrial fibrillation
DOAC: Direct oral anticoagulant
EBK: Empirical Bayesian Kriging
EHR: Electronic health record
GIS: Geographic Information System
INR: The international normalized ratio
IS: Ischemic stroke
TTR: Time in therapeutic range
WHO: World Health Organization
